# The HDAC6 inhibitor C1A modulates autophagy substrates in diverse cancer cells and induces cell death

**DOI:** 10.1038/s41416-018-0232-5

**Published:** 2018-10-15

**Authors:** Maciej Kaliszczak, Erich van Hechanova, Yunqing Li, Hibah Alsadah, Katarzyna Parzych, Holger W. Auner, Eric O. Aboagye

**Affiliations:** 1 Department of Surgery and Cancer, Cancer Imaging Centre, Imperial College London, Hammersmith Hospital, Du Cane Road, London, W12 0NN UK; 2 Cancer Cell Protein Metabolism Group, Department of Medicine, Imperial College London, Hammersmith Hospital, Du Cane Road, London, W12 0NN UK; 3 Present Address: Pre-clinical Imaging and Pharmacology, Biogen, 125 Broadway Street, Cambridge, MA 02142 USA; 4 Present Address: Developmental Biology of Birth Defects Section, UCL Great Ormond Street Institute of Child Health, University College London, 30 Guilford Street, London, WC1N 1EH UK

**Keywords:** Cancer therapeutic resistance, Predictive markers

## Abstract

**Background:**

Cytosolic deacetylase histone deacetylase 6 (HDAC6) is involved in the autophagy degradation pathway of malformed proteins, an important survival mechanism in cancer cells. We evaluated modulation of autophagy-related proteins and cell death by the HDAC6-selective inhibitor C1A.

**Methods:**

Autophagy substrates (light chain-3 (LC-3) and p62 proteins) and endoplasmic reticulum (ER) stress phenotype were determined. Caspase-3/7 activation and cellular proliferation assays were used to assess consequences of autophagy modulation.

**Results:**

C1A potently resolved autophagy substrates induced by 3-methyladenine and chloroquine. The mechanism of autophagy inhibition by HDAC6 genetic knockout or C1A treatment was consistent with abrogation of autophagosome–lysosome fusion, and decrease of Myc protein. C1A alone or combined with the proteasome inhibitor, bortezomib, enhanced cell death in malignant cells, demonstrating the complementary roles of the proteasome and autophagy pathways for clearing malformed proteins. Myc-positive neuroblastoma, KRAS-positive colorectal cancer and multiple myeloma cells showed marked cell growth inhibition in response to HDAC6 inhibitors. Finally, growth of neuroblastoma xenografts was arrested in vivo by single agent C1A, while combination with bortezomib slowed the growth of colorectal cancer xenografts.

**Conclusions:**

C1A resolves autophagy substrates in malignant cells and induces cell death, warranting its use for in vivo pre-clinical autophagy research.

## Introduction

Autophagy is a process for clearing malformed, damaged or superfluous proteins within the intracellular compartment into autophagosomes for delivery to lysosomes for degradation and recycling (Supplementary Fig. 1). While the context-specific role of autophagy in cancer is still debated, it is recognised that autophagy can serve two key functions: a tumour-suppressive function through elimination of oncogenic proteins, and perhaps for established cancer, a tumour-promoting function via recycling of metabolites to maintain mitochondrial functionality.^1^ It is the therapeutic role of autophagy-targeting drugs in cancer that is receiving recent attention due to the potential of such therapies to induce apoptosis or by-pass apoptosis defects to induce other forms of cytotoxicity.^2^ Enhanced autophagy can ensue following chemotherapy, and inhibition of autophagy under such conditions can lead to increased cell death as a cellular response to avoid accumulation of toxic proteins.^3,4^ Another important protein homeostasis mechanism in cells is governed by the proteasome. Proteasome inhibitors including bortezomib and carfilzomib are used clinically in multiple myeloma and selected other B cell malignancies, and are thought to exert their anti-tumour effects by triggering the accumulation of toxic misfolded proteins.^5^ Autophagy is widely thought to contribute to proteasome inhibitor resistance in myeloma by providing an alternative pathway for clearance of dysfunctional proteins. Cells can also curtail further accumulation of dysfunctional proteins through decreased protein translation via the unfolded protein response (UPR). The UPR is a highly conserved pathway that operates to prevent or correct a cellular phenotype termed endoplasmic reticulum (ER) stress via an adaptive response through a specific gene transcription programme; this response and autophagy are thought to control cell viability in cells where abnormal protein homeostasis persists.^6^

Unfortunately, to date, we have available only a limited number of inhibitors to study autophagy in vitro and in vivo. The anti-malarial agent chloroquine (CQ) that blocks lysosome acidification (Supplementary Fig. 1) has been evaluated pre-clinically and in patients alone and in combination with chemotherapy for autophagy inhibition.^7–9^ These studies have demonstrated that it is difficult to achieve robust autophagy inhibition at tolerable dose levels of CQ. Antagonists of the phosphoinositide 3-kinase-mammalian target of rapamycin pathway have also been tested as autophagy inhibitors. In particular, 3-methyladenine (3-MA; Supplementary Fig. 1) and 3-MA derivatives with improved solubility have been tested and show activity in vitro.^10^ Lastly, histone deacetylase 6 (HDAC6) inhibitors and pan-HDAC inhibitors have been evaluated as autophagy inhibitors. HDAC6 is involved in ubiquitin-dependent or ubiquitin-independent protein aggregate formation, as well as their clearance via autophagy.^11–14^ HDAC6, in association with the dynein motor complex, recruits and transports misfolded polyubiquitinated proteins via the microtubule network to aggresomes/autophagosomes for subsequent degradation by lysosomes.^13,15^ Pan-HDAC inhibitors combined with bortezomib are potent in resistant cancers due to the complementary roles of the autophagy and proteasome pathways in protein recycling; however, such combination is poorly tolerated clinically.^16^ It should be noted, however, that not all HDAC6 inhibitors can modulate autophagy. The lack of activity may be related to pharmacokinetics; the clinical HDAC6 candidate ricolinostat (ACY-1215) was recently reported to lack sufficient serum concentration to directly induce anti-multiple myeloma activity in vivo.^17^ However, at the cellular level, the HDAC6 inhibitor HPOB, while possessing potent HDAC6 deacetylase activity, does not modulate autophagy.^17,18^

We have previously described a selective HDAC6 inhibitor, C1A, with good pharmacokinetics and in vivo potency in solid tumour xenografts.^19^ In this work, we evaluated the effect of C1A on  autophagy and cell death in cancer subtypes presumed to be susceptible to autophagy inhibition (including cells harbouring mutant KRAS, Myc protein, or cells that produce high levels of immunoglogulin and are dependent on efficient clearance of cytotoxic misfolded protein aggregates for survival).^1,20,21^

## Materials and methods

### Compounds

C1A was synthesised as previously described.^19^ Suberoylanilide hydroxamic acid (SAHA) and tubastatin A were purchased from Cayman Chemical (Ann Arbor, MI, USA). 3-MA and chloroquine were from Sigma (St Louis, MO, USA). Bortezomib was from EMD Millipore (Nottingham, UK). ACY-1215 was purchased from Selleckchem (Munich, Germany).

### Antibodies

Antibodies against active cleaved caspase-3, C-Myc, HDAC6, LC3B and N-Myc, acetyltubulin and tubulin, acetyl-H3 and H3 were from Cell Signalling (Beverly, MA, USA). Anti-β-actin was from Abcam (Cambridge, UK).

### Cells

Colorectal  cancer HCT116 cells were obtained from ATCC. Osteosarcoma U2OS cells transfected with light chain-3B (LC3B) were a gift from Prof. Michael Seckl (Imperial College London, London, UK). Mouse embryonic fibroblasts (MEFs) proficient (WT) and deficient (KO) in HDAC6 were kindly provided by Prof. Tso-Pang Yao (Duke University, Durham, NC, USA). We have previously characterised these cells.^22^ Multiple myeloma cells were obtained from the American Type Culture Collection or Deutsche Sammlung von Mikroorganismen und Zellkulturen. Neuroblastoma cells were a gift from Prof. Louis Chelser (The Institute for Cancer Research, London, UK). The Tet21/N cells were generated by stable transduction of SHEP cells with a Tet-off (tetracycline withdrawal-inducible) N-Myc expression construct.^23^ TGR-1 (*Myc*+/+, *Myc−/−* and *Myc−/−*wtMyc) rat fibroblast cell lines were kindly provided by Prof. Prochownik (University of Pittsburgh School of Medicine, Pittsburgh, PA, USA). These cells have previously been characterised for Myc expression.^24^ All cells were passaged in our laboratory for fewer than 6 months on receipt and were tested mycoplasma free.

### Immunofluorescence and p62 ELISA

Exponentially growing HCT116 cells and osteosarcoma U2OS-LC3-GFP cells were seeded in chamber slides (Sigma) on day 1. The cells were treated on day 2 with different compounds for 24 h and stained by immunofluorescence as described elsewhere.^25^ The mCherry-GFP-LC3 construct was a generous gift from Dr. Terje Johansen (University of Tromsø, Tromsø, Norway). MEFs were transfected with the plasmid using Lipofectamine 2000 (Invitrogen, Thermo Fisher, Waltham, MA, USA) for 24 h before treatment with C1A. Cells were subsequently fixed with 4% paraformaldehyde, washed and visualised with a Olympus BX51 microscope (Olympus UK Ltd, London, UK). Positive cells were counted using the ImageJ software (NIH, Bethesda, MD, USA) and expressed as a percentage of total cells counted. Autophagy markers p62 were determined by enzyme-linked immunosorbent assay (ELISA) (kit from Enzo life Sciences, Farmingdale, NY, USA).

### ER stress quantitation by real-time PCR

KELLY cells were treated continuously with reagents or vehicle for 24 or 72 h. At the end of the incubation period both adherent and detached cells were pelleted, washed with phosphate-buffered saline and frozen at −80 °C prior to analysis. Transcripts were analysed after reverse transcription by real-time quantitative PCR as previously described.^26^ Results are shown as fold change in mRNA expression of the gene of interest, relative to *GAPDH* compared to the relevant vehicle-treated control condition.

### Caspase-3/7 assay

Caspase-3/7 activity was determined using Promega’s caspase-3/7 assay according to the manufacturer’s instructions (Promega, Madison, WI, USA). Briefly, cells were transferred in a white opaque 96-well plate, incubated for 1 h with Caspase-Glo reagent and the enzymatic activity of caspase-3/7 was measured using a TopCount NXT microplate luminescence counter (PerkinElmer, Waltham, MA, USA). To enable normalisation of data to total cellular protein content, the sulforhodamine B (SRB) assay was performed in parallel for all samples.^27^

### ATPlite measurement assay

Suspension cells were seeded into white, clear-bottom 96-well plates for 24 h and subsequently treated with C1A, ACY-1215, bortezomib alone or in combination for 24 h. One hundred microliters of ATPlite luminescence (PerkinElmer) reagent was added and luminescence was measured using a TopCount NXT microplate luminescence counter (PerkinElmer).

### Tumour xenografts

HCT116 (5 × 10^6^) and KELLY (7.5 × 10) cells were injected subcutaneously in 100 and 150 µL volumes, respectively, into the flank of female nu/nu-BALB/c athymic nude mice (Harlan, UK). Tumour measurements were performed every day and volumes were calculated using the formula [length (mm)] × [width (mm)] × [depth (mm)] × *π*/6. The animals were randomised, and when tumours had reached a volume of 50–100 mm,^3^ animals were entered into the different treatment groups and treatment with different compounds was initiated (C1A, bortezomib alone or in combination). Animals were treated intraperitoneally (C1A and bortezomib) for a total of 14 days. Both compounds were dissolved in dimethyl sulfoxide (DMSO) (10%) and 0.9% saline. Throughout the 14-day treatment period, animal weights and tumour volumes were determined each day. All animal experiments were done by licensed investigators in accordance with the United Kingdom Home Office Guidance on the Operation of the Animal (Scientific Procedures) Act 1986 (HMSO, London, UK, 1990) and within guidelines set out by the United Kingdom National Cancer Research Institute Committee on Welfare of Animals in Cancer Research.^28^

### Cell viability and cytotoxicity assays

Drug concentrations that inhibited 50% of cell growth (GI_50_) for adherent cell lines were determined using an SRB technique. All cell lines were treated for 24 h on day 2 and allowed to grow for an additional 3 days. Optical densities were measured at 540 nm with a Multiskan EX photometer from Thermo Electron Corporation using the Ascent Software version 2.6 (Thermo Labsystems Oy, Vantaa, Finland). Growth inhibition curves were plotted as the percentage of control cells and GI_50_ values were determined by the GraphPad Prism 5 Software (San Diego, CA, USA) by fitting a sigmoidal curve with variable slope.

### Immunoblotting

Cells and tumour tissue samples were prepared and subjected to western blotting as previously described.^29^ All primary antibodies were used at 1/1000 apart from anti-β-actin, which was diluted at 1:10,000. Anti-horseradish peroxidase-conjugated antimouse, anti-rabbit and anti-rat IgG secondary antibodies were used at a concentration of 1:2000. Densitometry data from western blot experiments were generated using the ImageJ software (NIH, USA) by measuring grey intensities of lanes corresponding to the protein of interest relative to the intensity of the lane corresponding to the loading control. Data are expressed as a fold change compared to control. It is indeed possible that some densitometry data (fold increase) were overestimated in cases where control values were nearly undetectable. This method is semi-quantitative and absolute data should indeed be interpreted with caution. Where available (e.g. measurements of p62) quantitative ELISA was used.

### Statistical analyses

*T* test or one-way analysis of variance with Dunnett’s multiple comparisons test was used for analyses, which were performed using the GraphPad prism software (GraphPad, La Jolla, CA, USA), and *P* values <0.05 using a 95% confidence interval were considered significant. Data are reported as mean ± s.e.m. of at least three independent experiments, unless otherwise stated. *P < 0.05, **P < 0.005 and ***P < 0.0001 were considered to be statistically significant; NS, not significant.

## Results

### HDAC6 inhibition by C1A modulates autophagy

**Fig. 1:**
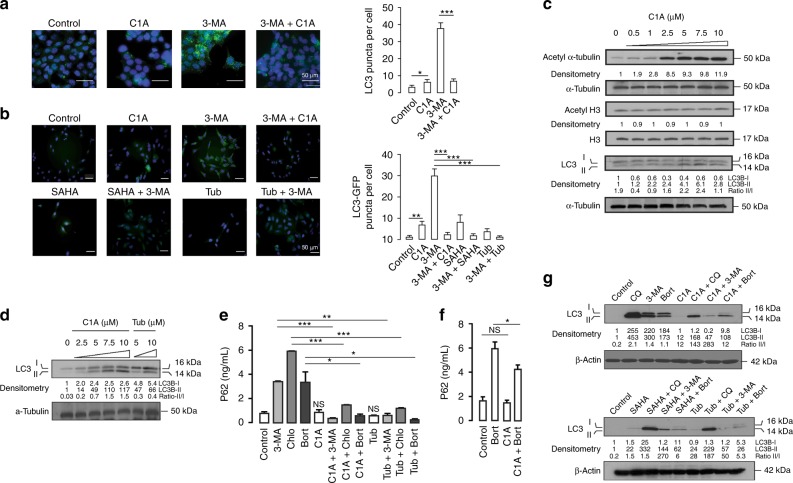
C1A impairs autophagic maturation in colorectal cancer HCT116 cells and osteosarcoma U2OS-LC3-GFP cells. **a**, **b** Effect of C1A (10 μM) on 3-MA (5 mM; 24 h) induced increase of LC3 puncta (green) detected by immunofluorescence in HCT116 cells (**a**) and in U2OS-LC3-GFP cells (**b**). DNA (blue) was stained with DAPI. Treatment with SAHA and tubastatin A (Tub) are shown for comparison. Bar = 50 µm in each image. Summary data for counted puncta are shown next to the images; >100 cells were scored for each sample (*n* = 3). **c**, **d** Western blots of LC3 following 24 h treatment with C1A in HCT116 cells (**c**) and U2OS-LC3-GFP cells, stably transfected with LC3-GFP (**d**). The lower band corresponds to LC3B-II marker of autophagosome formation. Treatment with tubastatin A is shown for comparison. Acetylation of α-tubulin was used as a marker of HDAC6 inhibition (vs. acetylation of histone H3, HDAC class I inhibition). ‡Note that absolute LC3-II/LC3I ratios are presented. **e** Levels of p62, a marker of autophagosome formation, as evaluated by ELISA in HCT116 cells. **f** Effect of C1A on bortezomib-induced increase of p62 levels in U2OS-LC3-GFP cells as determined by ELISA. **g** Effect of C1A on LC3 induction by autophagy tool compounds. HCT116 cells were treated with 3-MA (5 mM), chloroquine (CQ- 50 μM), bortezomib (Bort, 5 nM), SAHA (10 μM), tubastatin A (10 μM) and C1A (10 μM) alone or in combination for 24 h and assessed by western blot. Blots are representative of three independent experiments. Results are representative of three independent experiments performed in triplicate (mean ± SEM). NS, not significant compared to control. *P < 0.05, **P < 0.005, ***P < 0.0001

We induced autophagy and assessed changes in autophagy substrates under complete growth media conditions (quality control autophagy) following C1A treatment (10 μM, 24 h). Treatment of colorectal cancer HCT116 cells (KRAS exon 2: G13D heterozygously mutated allele) with 3-MA (5 mM, 24 h) increased microtubule-associated protein-1 LC3 that is localised on autophagosomal membrane during autophagy (Fig. 1a).^30,31^ LC3 puncta determined by immunofluorescence (as green fluorescence using anti-LC3B antibody, D11) increased by 13-fold following 3-MA, an effect that was abrogated following co-incubation with C1A. To confirm specificity of the process, we repeated the experiments in osteosarcoma U20S-LC3-GFP cells; the LC3-GFP fused protein is often used to detect autophagy through increased cytoplasmic GFP puncta. Again, C1A resolved GFP puncta induced by 3-MA (Fig. 1b). The pan-HDAC inhibitor SAHA (10 μM, 24 h) and the HDAC6-selective tool compound, tubastatin A (10 μM, 24 h), also resolved 3-MA-induced autophagy under similar conditions. Small increases in LC3 were seen by western blot at concentrations of C1A alone that affected HDAC6 catalytic function as demonstrated by acetylation of acetyl-α-tubulin but not acetyl-H3 (Fig. 1c, d); in this case, LC3I degradation that would be suggestive of enhanced ‘autophagic flux’ was not observed. Finally, changes in p62 expression can serve as a useful marker for the induction of autophagy. Increased p62 level can indeed represent a blockade of autophagy, and conversely, activation could be demonstrated by decreases in p62 levels.^32,33^ Notably, however, p62 can also be transcriptionally regulated by autophagy.^34^ Under the short-term treatments used here, C1A and tubastatin A suppressed increases in 3-MA-induced, chloroquine-induced or bortezomib-induced p62 expression determined by ELISA (Fig. 1e, f).

Western blot analysis of LC3 was in keeping with the p62 assessment by ELISA (Fig. 1g). From the foregoing, it appears that C1A by itself mildly induces expression of LC3, but more importantly, is potent at resolving LC3 induced by 3-MA.

Given the published role of HDAC6 in transporting malformed proteins along dynein motors to the lysosomes, we determined whether C1A suppressed autophagosome fusion with lysosomes using a tandem tagged LC3-GFP-mCherry probe previously described by Ganley et al.^35^

**Fig. 2:**
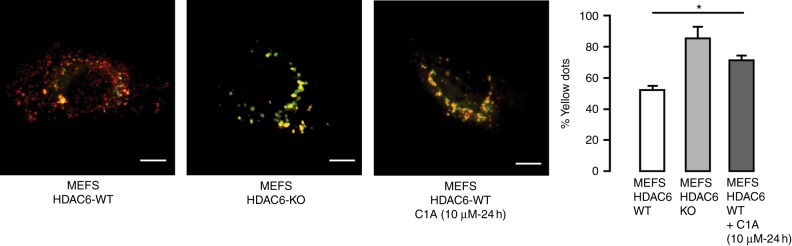
HDAC6 inhibition by C1A prevents autophagosome–lysosome fusion. MEFS with different status of HDAC6 (WT vs. KO) were transfected with plasmid pH-sensitive LC3-tagged plasmid with GFP and mCherry and yellow spots quantified. GFP tag (green) is degraded in acidic conditions (e.g. found in lysosomes), whereas mCherry tag is stable (red). Yellow spots (mix of red and green) show lack of acidification. Comparison was done with treatment with C1A for 24 h at 10 μM. Bar = 2 µm in each image. Summary data for counted puncta are shown next to the images; >100 cells were scored for each sample (*n* = 3)

While the low pH of the lysosome quenches GFP fluorescence, both GFP (green) and mCherry (red) fluoresce under the near-neutral pH outside the lysosome (Fig. 2). We found that MEFs transfected with the probe under complete media conditions show mainly mCherry fluorescence consistent with autophagosome fusion with lysosomes and GFP quenching. In contrast, MEFs in which HDAC6 has been genetically knocked out showed green and yellow fluorescence consistent with inhibition of fusion and this outcome was phenocopied by C1A treatment, demonstrating that C1A prevents autophagosome fusion with lysosomes.

### C1A synergises with bortezomib in vitro and slows tumour growth in vivo

**Fig. 3:**
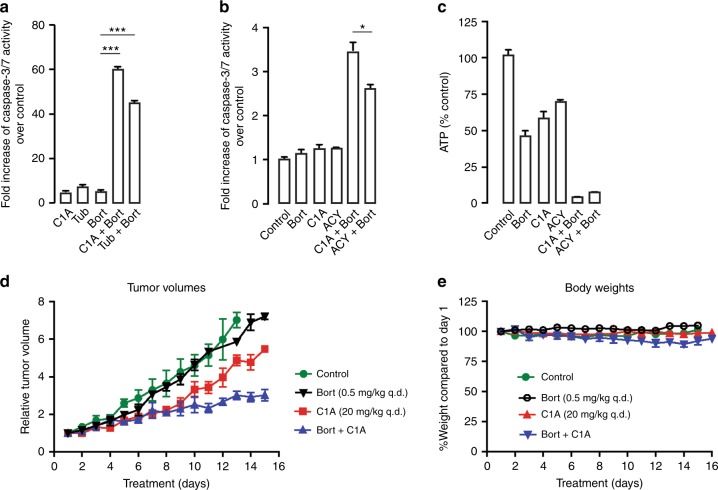
HDAC6 inhibitor C1A synergises with proteasome inhibitor in vitro and in vivo. **a** Effect of 24 h treatment with bortezomib (Bort, 5 nM) alone or in combination with C1A (10 µM) or tubastatin A (Tub, 10 µM) on caspase-3/7 activity in HCT116 cells. **b**, **c** Effect of 9 h treatment with bortezomib (Bort, 5 nM) alone or in combination with C1A (2 µM) or ACY-1215 (ACY, 2 µM) on caspase-3/7 activity (**b**) and intracellular ATP levels (**c**) in OPM-2 cells. **d** Anti-tumour activity of bortezomib (Bort) alone or in combination with C1A in HCT116 xenograft model. Both C1A and bortezomib were given intraperitoneally once daily (q.d.). **e** Corresponding body weights during the time course of the study. Results are representative of three independent experiments performed in triplicate (mean ± SEM). NS, not significant compared to control. *P < 0.05, **P < 0.005, ***P < 0.0001.

Towards clinical application, the pan-HDAC inhibitor, vorinostat (SAHA), has been shown to synergise with bortezomib to induce apoptosis in colorectal cancer cells via an autophagic mechanism.^36^ Surmising that this cytotoxicity occurred via an HDAC6 function, we investigated the ability of C1A to induce caspase-3/7-dependent cell death consequent to bortezomib-enhanced autophagy flux. C1A or bortezomib single treatment increased caspase-3/7 activity in HCT116 colorectal cells by 6-fold or 7-fold, respectively, over control. In contrast, the combination of C1A and bortezomib synergistically increased caspase-3/7 activity an order of magnitude higher (Fig. 3a). In addition, tubastatin A similarly synergised with bortezomib in the colon cancer cells. Bortezomib is approved for use in multiple myeloma and the HDAC6-selective inhibitor, ACY-1215, was shown to synergise with bortezomib in MM.1S and RPMI8226 myeloma cells,^37^ and as opposed to single agent showed 37% clinical response rate in multiple myeloma patients.^38^ In this report, we show that either C1A or ACY-1215 at equimolar concentrations synergises with bortezomib to induce caspase-3/7 activity in OPM-2 multiple myeloma cells (Fig. 3b). At the low micromolar concentrations of the drugs, near-complete loss of ATP accompanied the increase in caspase-3/7 activation (Fig. 3c), perhaps indicating a mechanism by which these compounds affect cell death, although this aspect needs further studies. Tubastatin A has limited activity in vivo due to poor pharmacokinetics. The potent and sustained activity of C1A relative to ACY-1215 under ‘pulsed treatment-drug washout’ conditions that mimic in vivo varying drug concentrations (Supplementary Fig. 2), and its demonstrated activity in vivo in solid tumours, makes C1A an ideal tool compound.^19,22^

HCT116 colon cancer cells reproducibly form tumours in immune-deficient mice and we have previously determined the efficacy of C1A in this model in vivo.^19,22^ From the foregoing, we hypothesised that C1A will potentiate the effect of bortezomib in HCT116 xenografts. Bortezomib was minimally effective in HCT116 xenografts (Fig. 3d). C1A slowed tumour growth and the combination of C1A and bortezomib was more potent than either drug alone (Fig. 3d). The combination was well tolerated in vivo with no significant change in body weight of mice (Fig. 3e).

### Exploring single agent activity of C1A in cell lineages with high Myc expression

While the autophagy-modulating effects of different drug combinations have been reported, a deficiency in our current understanding of how to use HDAC6-selective inhibitors is the genetic basis to select patient populations that would be enriched for therapeutic response. KRAS-driven tumours and B cell lineage tumours, such as multiple myeloma (dependent on clearing high levels of immunoglobulin), have been suggested (vide supra).^1,20^ It would appear, however, that malignant cells with high expression of an oncogene that drives high levels of protein synthesis would be a candidate as cells generally lack efficient mechanisms to curtail superfluous protein synthesis and are thus dependent on the proteasome/autophagosome to ride it of potentially toxic protein. Myc, an oncoprotein that is frequently genetically amplified or whose transcriptional regulation is aberrant, potently activates protein synthesis via eukaryotic initiation factor 4F (eIF4F), the key regulator of the mRNA–ribosome recruitment phase of translation initiation.^39^ Indeed, in multiple myeloma cells, Myc has been shown to critically regulate aggresome/autophagosome formation and apoptosis in response to bortezomib and SAHA.^40^ Recently, Belounis et al.^21^ showed that autophagy is present in neuroblastoma cells, induced by chemotherapy and associated with chemoresistance. These studies warrant assessment of HDAC6 inhibitors in Myc-dependent cancers including B cell lineage (C-Myc-driven) and neuroblastoma (N-Myc-driven).

**Fig. 4:**
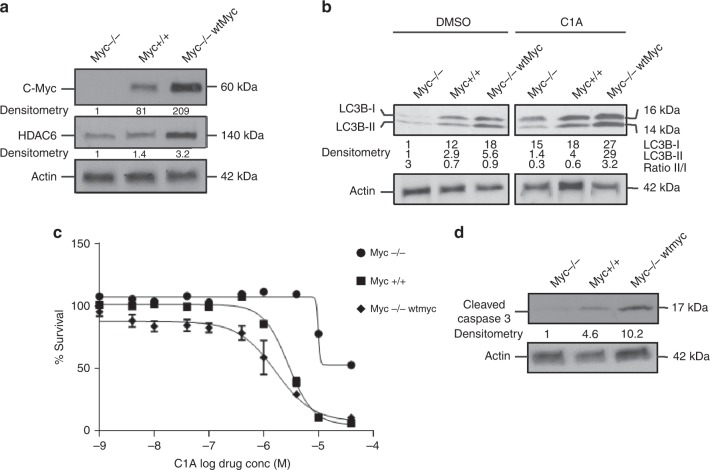
Reduced expression of Myc abrogates the ability of HDAC6i to resolve autophagic response in TGR-1 rat fibroblasts. A cell line with endogenous levels of Myc. (*Myc+/+*), an isogenic line bearing a homozygous deletion of Myc (*Myc−/−*), and *Myc−/−* cells stably transduced with a lentiviral vector encoding wild-type human Myc (*Myc−/−*wt*Myc* cells) were used in this study. **a** Western blot showing relative levels of HDAC6 in cell lines with different Myc expression. **b** Relative expression of LC3 following treatment with C1A (10 μM) or DMSO for 24 h. **c** Growth inhibitory effect of C1A over 72 h of treatment. Results are expressed as a percentage of control cells. **d** Impact of C1A following 24 h treatment with C1A at 10 μM on cleaved caspase-3, as a marker of apoptosis

Rat fibroblast cell lines with endogenous levels of Myc (*Myc*+/+), an isogenic line bearing a homozygous deletion of Myc (*Myc−*/*−*), and Myc*−*/*−* cells stably transduced with a lentiviral vector encoding wild-type human Myc (*Myc−*/*−*wt*Myc*) showed differential C-Myc expression (Fig. 4a).^24^ HDAC6 expression (using rat HDAC6 antibody) was higher in *Myc−*/*−*wt*Myc* and *Myc*+/+ cells compared to *Myc−*/*−* cells (Fig. 4a).

LC3 expression, cell growth inhibition by C1A and C1A-induced caspase-3/7 activation all increased in the order *Myc−*/*−* cells < *Myc*+/+ cells < *Myc−*/*−*wt*Myc*, consistent with C-Myc-dependent autophagy and sensitivity to HDAC6 inhibition (Fig. 4b, c).

**Fig. 5:**
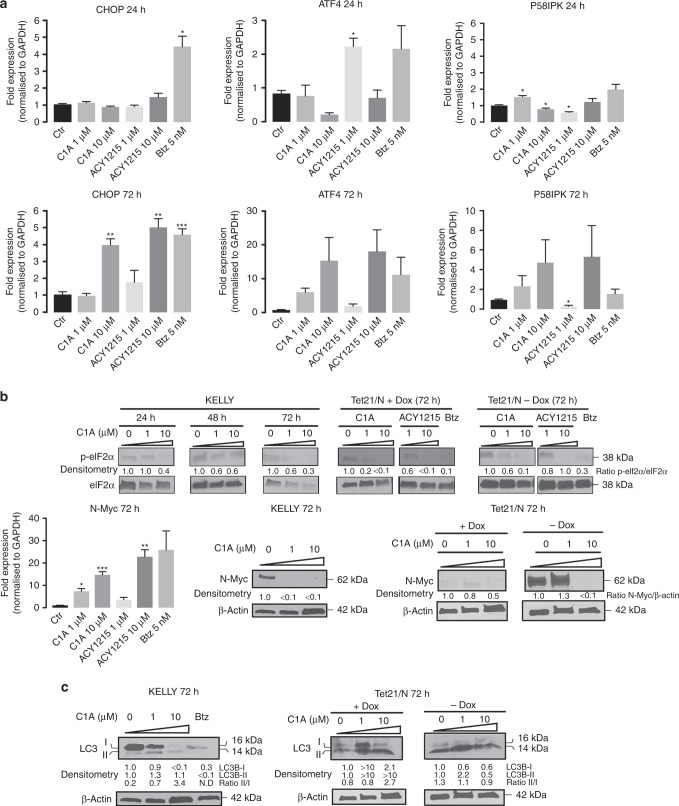
Effect of HDAC6 inhibitors on ER stress and protein recycling in N-Myc high and low cells. **a** Effect of HDAC6 inhibitors on ER stress. N-Myc high KELLY cells were treated continuously with vehicle, C1A (1, 10 µM), ACY-1215 (1, 10 µM) or bortezomib (Btz, 5 nM) for 24 (acute response) or 72 h (chronic response) and samples were analysed by real-time quantitative PCR for *CHOP*, *ATF4* and *P58IPK* mRNA. **b** Effect of HDAC6 inhibitors on p-eIF2α/eIF2α protein expression (top panel) and *N-Myc* mRNA or protein expression (lower panel) in KELLY cells or in N-Myc-high Tet21/N (−Dox) and N-Myc-low Tet21/N cells (+Dox) cells. The high cell death observed with C1A-treated KELLY cells at 72 h may account for the inconsistent density of eIF2α seen across the stated concentrations. Densitometry of ratios p-eIF2α/ eIF2α and N-Myc/ β-actin of the treated groups were normalised to the untreated at respective time points. Dox, 2 µg/mL doxycycline. **c** Effect of HDAC6 inhibitors on LC3I and LC3-II proteins. ^‡^Note that absolute LC3-II/LC3I ratios are presented. Dox, 2 µg/mL doxycycline. Results are representative of three independent experiments performed in triplicate (mean ± SEM). NS, not significant compared to control. *P < 0.05, **P < 0.005, ***P < 0.0001.

While these results suggests that Myc may play a role in sensitivity to HDAC6 inhibition, the exact mechanisms are still unclear. We therefore investigated the effects of C1A and ACY-1215 on ER stress and Myc. Both inhibitors triggered limited increases in *CHOP*, *ATF4* and *P58IPK* mRNA—components of the UPR and thus indicators of ER stress—at 24 h in Myc high KELLY cells, suggesting that the changes in caspase-3/7 seen at this time point were largely independent of ER stress (Fig. 5a). A  concentration-dependent induction of *CHOP*, *ATF4* and *P58IPK* mRNA expression at 72 h demonstrate that C1A and ACY-1215 caused a late-onset transcriptional ER stress response. However, we could not detect increased phosphorylation of eIF2α^Ser51^ (Fig. 5b), an upstream biomarker of UPR activation, early (24 h) or late (48–72 h) after C1A or ACY-1215 treatment. In fact, C1A and ACY-1215 treatment resulted in decreased eIF2α phosphorylation in KELLY cells and in Tet21/N cells. In the latter cells, the reduction in eIF2α phosphorylation appeared to be largely independent of whether N-Myc was expressed at low (+Dox) or high (*−*Dox) levels. Thus, C1A and ACY-1215 induce an atypical and N-Myc-independent late-onset ER stress response. Interestingly, *N-Myc* mRNA expression was increased by C1A, whereas N-Myc protein levels were virtually undetectable in KELLY cells upon C1A treatment (1 and 10 μM) and in Myc -low Tet21/N cells (10 μM C1A; Fig. 5b). We also observed that C1A led to a reversal in the LC3B-II/I ratio in KELLY cells, with an overall increase in detectable LC3B-II/I on immunoblots. In Tet21/N cells, the effects of 10 μM C1A on LC3B-II/I were comparable in N-Myc high and low cells, but appeared different at the lower dose of 1 μM (Fig. 5c). Taken together, C1A downregulates N-Myc protein levels despite persistent transcriptional induction, and its effects on autophagic processes appear to be partly linked to N-Myc expression. The precise mechanisms by which C1A treatment regulates N-Myc, and how their activity relates to autophagy and the UPR, remain to be established but are likely to be biologically relevant.

**Fig. 6:**
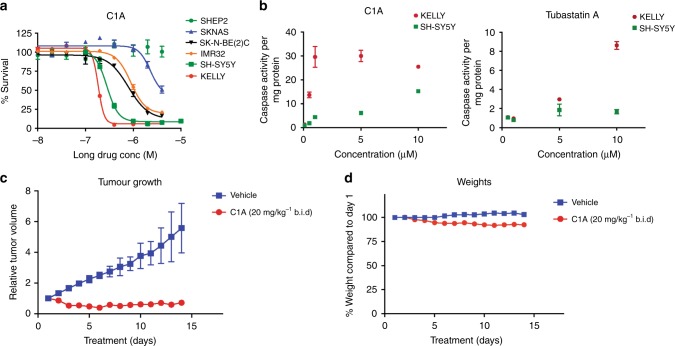
Myc-proficient neuroblastoma cells are sensitive to C1A. **a** Growth inhibitory effect of C1A in a panel of neuroblastoma with different N-Myc expression. **b** Impact of HDAC6 inhibitors, C1A and tubastatin A, on caspase- 3/7 activity in SH-SY5Y and KELLY cells. Data are normalised by the amount of protein. Cells were treated for 24 h at the indicated concentration. **c**, **d** In vivo anti-tumour activity (**c**) of C1A and corresponding body weights (**d**) in KELLY mouse model of neuroblastoma. C1A was given twice daily at 20 mg/kg for 14 days (*n* = 3)

HDAC6-selective inhibitors C1A and ACY-1215 were potent in multiple myeloma cell lines (ARH77, JJN3, KMS12, U266, RPMI8226, KMS11, OPM-2) with a mean GI_50_ of 0.48 and 1.04, respectively (Supplementary Table 1). In neuroblastoma cells (KELLY, SH-SY5Y, SHEP, SKNAS, SK-N-BE(2)C, IMR32, SKNSH), the GI_50_ for C1A ranged between 0.18 and 16; with SAHA, the range was narrower (0.35 and 1.4) (Supplementary Table 1). From previous reports, we could classify neuroblastoma cell lines with no or low N-Myc expression (SHEP, SKNAS), with N-Myc amplification (IMR32, SK-N-BE(2)C and SH-SY5Y) and with high N-Myc amplification (KELLY).^41–44^ Sensitivity of the neuroblastoma cell lines to C1A was found to be dependent on N-Myc protein expression, with KELLY being the most sensitive cell line (Fig. 6a). Using tubastatin A as a positive control, we showed that caspase-3/7 activation following HDAC6-selective C1A treatment of two neuroblastoma cell lines was inhibitor type and concentration dependent (Fig. 6b). Given the sensitivity of Myc-positive cells to C1A, we correlated C1A sensitivity in the NCI60 cell line panel to Myc expression. There was no correlation between C1A sensitivity and C-Myc or *N-Myc* mRNA expression (Supplementary Fig. 3), indicating that *Myc* mRNA expression per se does not represent a robust biomarker of C1A sensitivity ordinarily or in the context of autophagy.

From the sensitivity screen and N-Myc protein expression data, we selected the KELLY neuroblastoma cell line for further testing in vivo. Daily injection of single agent C1A at 20 mg/kg b.i.d. led to profound tumour growth arrest in the KELLY xenografts without any effect on mouse body weight (Fig. 6c, d).

## Discussion

This study shows that the hydroxamate-based small-molecule C1A can phenocopy HDAC6 inhibition and modulate autophagy in cancer cells from different origins. Markers of autophagy (i.e., LC3 and p62) were indeed altered following treatment in colon cancer and osteosarcoma cells. Not discounting other mechanisms of cell death, C1A was shown to induce apoptosis in those cells in the same range of concentrations and it is possible that autophagy served as a survival mechanism that when abrogated led to cell death. Markers of autophagy (e.g. the  increased expressions of LC3BII and LC3BI in HCT116), however, suggested a blockade of the autophagy mechanism rather than an increase in the flux. We have also demonstrated in MEFs that C1A could phenocopy HDAC6 genetic knockout and alter the fusion of the autophagosomes and lysosomes. Autophagy has indeed been originally considered to be a non-specific bulk pathway by which cells scavenge cytoplasmic proteins and organelles in response to starvation.^45^ Other extracellular or intracellular stress signals such as growth factor deprivation, ER stress and pathogen infection were also shown to induce autophagy.^46^ The effects of C1A on apoptosis may be ER stress unrelated. C1A could potentially be used to modulate apoptosis and ER stress, and further applications are currently under investigation. Targeting protein homeostasis, both proteasomal and autophagy pathways were previously used as a strategy to treat nonsolid tumours. Hideshima et al.^47^ paved the way by using tubacin and bortezomib in pre-clinical models of multiple myeloma. HDAC6 inhibitor ACY-1215 has progressed to phase I/II clinical trials, but was associated with unfavourable pharmacokinetic properties.^17,38^ This outcome was potentially resolved with orally bioavailable compound WT161 that showed promise in combination with bortezomib in a pre-clinical model of multiple myeloma.^17^ To our knowledge, our study is the first to describe a strategy of tackling protein homeostasis in order to treat solid tumours. C1A synergised with bortezomib in colon cancer cells, greatly enhancing caspase-3/7 activation and was able to reduce tumour growth in  mouse models of disease.

We have shown that tumour types, with high protein turnover in general could be more susceptible to therapy with HDAC6 inhibitors. We have exemplified our findings with KRAS-positive colon cancer cells, Myc-amplified neuroblastoma (N-Myc-driven) and confirmed sensitivity of combination therapy in B cell lineage (C-Myc-driven). We were however not able to correlate sensitivity of C1A more broadly in the NCI60 panel of cell lines with C-Myc or N-Myc expression. Other than lineage dependence, it is possible that expression of Myc per se may be insufficient to predict sensitivity to HDAC6 inhibitors in all cell types. Myc transcriptional activity may lie in its ability to couple with binding partners (e.g. MAX) and form functional complexes.^48^ Myc activity can also be antagonised by the presence of MAD1 that acts as a transcriptional repressor.^49^ Upstream regulators could also affect the activity of Myc. For instance, *RAS* mutation was shown to contribute to the oncogenic role of N-Myc.^50^ The tyrosine kinase SRC has also been implicated in mediating growth factor signal-induced C-Myc expression.^51,52^ Even though a complex scenario likely exists for generation of superfluous protein requiring degradation—with Myc being a player—we can speculate that tumours would be primed to HDAC6 inhibitors as demonstrated in this report.^53^

We observed an elevated HDAC6 expression in fibroblasts overexpressing C-Myc, which is consistent with previous reports. For instance, Nawrocki et al.^40^ have shown, in human foreskin fibroblasts transfected with Myc, increased protein synthesis and aggresome/autophagosome formation alongside increased HDAC6 expression, suggesting that further that HDAC6 is a direct Myc transcriptional target,^40^ although our current work in neuroblastoma cells showed that C1A increased *Myc* mRNA level and decreased  its expression level. Another report by Lwin et al.^54^ linked C-Myc and HDAC6 expression in cell lines and primary lymphoma samples of mantle cell lymphomas and other B cell lymphomas. The authors provided evidence that activation of HDAC6 may be modulated by the microenvironment. This suggests that HDAC6 expression could also be predictive of sensitivity to HDAC6 inhibitors, but we previously showed no correlation between sensitivity to C1A and HDAC6 expression in the NCI60 panel.^19^ This might not seem surprising as the cells were tested under 'basic' conditions, which emphasises the necessity to develop and test more refined in vitro and primary tumour models that would mimic more closely an in vivo microenvironment.^55^ Such refinement would help ascertain the influence of Myc activity on HDAC6 expression, and further, prediction of HDAC6 inhibitors' sensitivity in numerous tumour types.

To conclude, we have shown that pharmacological inhibition of HDAC6 by C1A resolves autophagy induced by 3-MA and is cytotoxic to tumour cells alone or in combination with a proteasome inhibitor. We showed that the combination is more efficacious in vivo in nonsolid and solid tumours, warranting its use for in vivo pre-clinical autophagy research. Finally, we identified Myc expression (in neuroblastoma) as worthy of further evaluation as a predictive marker of response.

## Electronic supplementary material

Supplementary Figures

## Availability of data and material

All datasets on which the conclusions of the paper rely will be available to readers. Materials could be requested by email.
