# Supplementary material for: The HDAC6 inhibitor C1A modulates autophagy substrates in diverse cancer cells and induces cell death
Source: Br J Cancer. 2018 Oct 15;119(10):1278–87. doi: 10.1038/s41416-018-0232-5 (PMC6251030; doi:10.1038/s41416-018-0232-5)
Supplement: Supplementary file 1 — Supplementary Figures [file 41416_2018_232_MOESM1_ESM.docx]

­

**Supplementary Figures.**

**
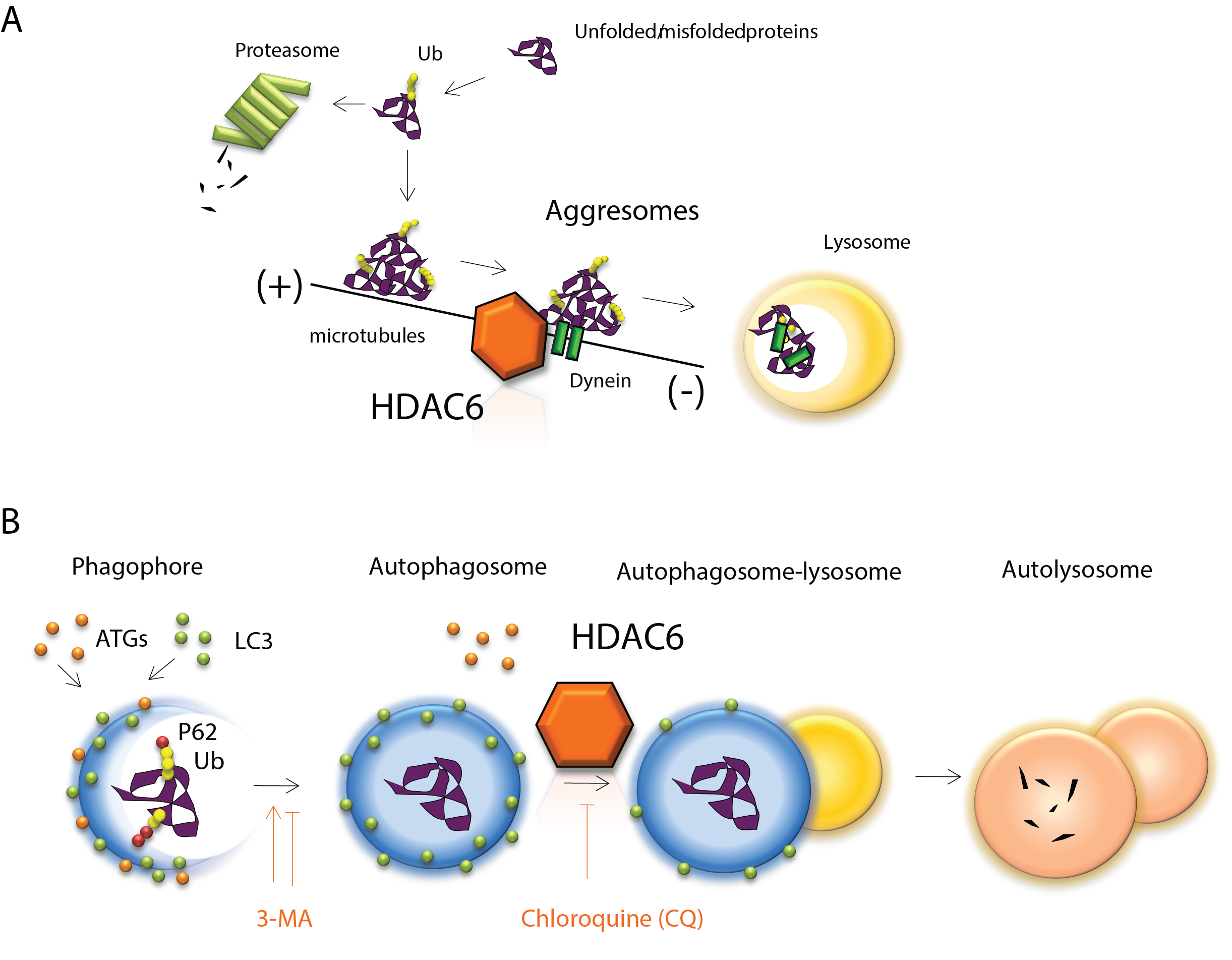
**

C

**
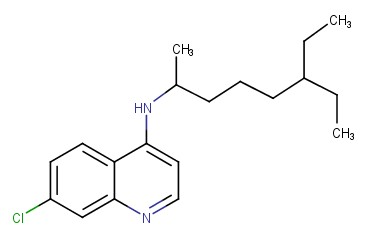
**Chloroquine
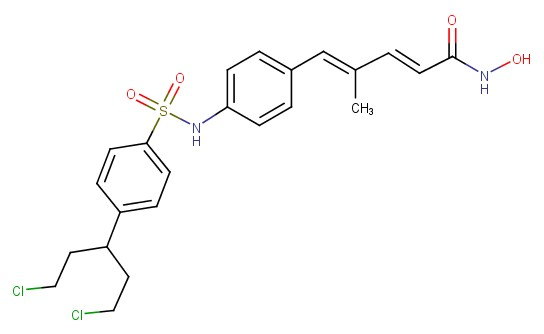
 C1A
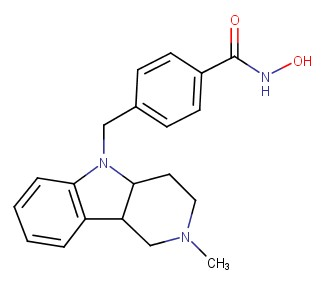
 Tubastatin A **
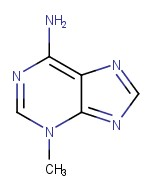
** 3-MA
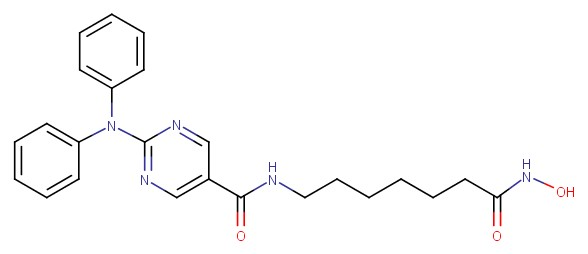
 ACY-1215

**Supplementary Fig. 1. Diagram depicting the roles of HDAC6 in protein degradation. A.** HDAC6 regulates aggresome formation and cell viability in response to misfolded protein stress. Kawagushi and coworkers have shown that HDAC6 has indeed the capacity to bind both polyubiquitinated misfolded proteins and dynein motors, thereby acting to recruit misfolded protein cargo to dynein motors for transport to aggresomes, thus maintaining protein homeostasis (Kawaguchi *et al*, 2003). **B.** HDAC6 participates in autophagy by controlling the fusion of autophagosomes to lysosomes (Lee *et al*, 2010). Upon signal, polyubiquitinated protein are tagged by P62, recruiting ATGs and LC3 to form autophagosomes. Small molecule 3-MA, is an inhibitor of phosphatidylinositol 3-kinases and was shown to be is a key regulator of autophagy and formation of autophagosomes. Both Inhibitory and stimulatory effects were reported (In quality control versus nutrient deprived autophagy). Chloroquine, an anti-malarial chemical, raises the lysosomal pH, inhibits fusion of lysosomes with autophagosomes and ultimately blocks autophagy. **C.** Chemical structures of modulators of autophagy used in this study.

**
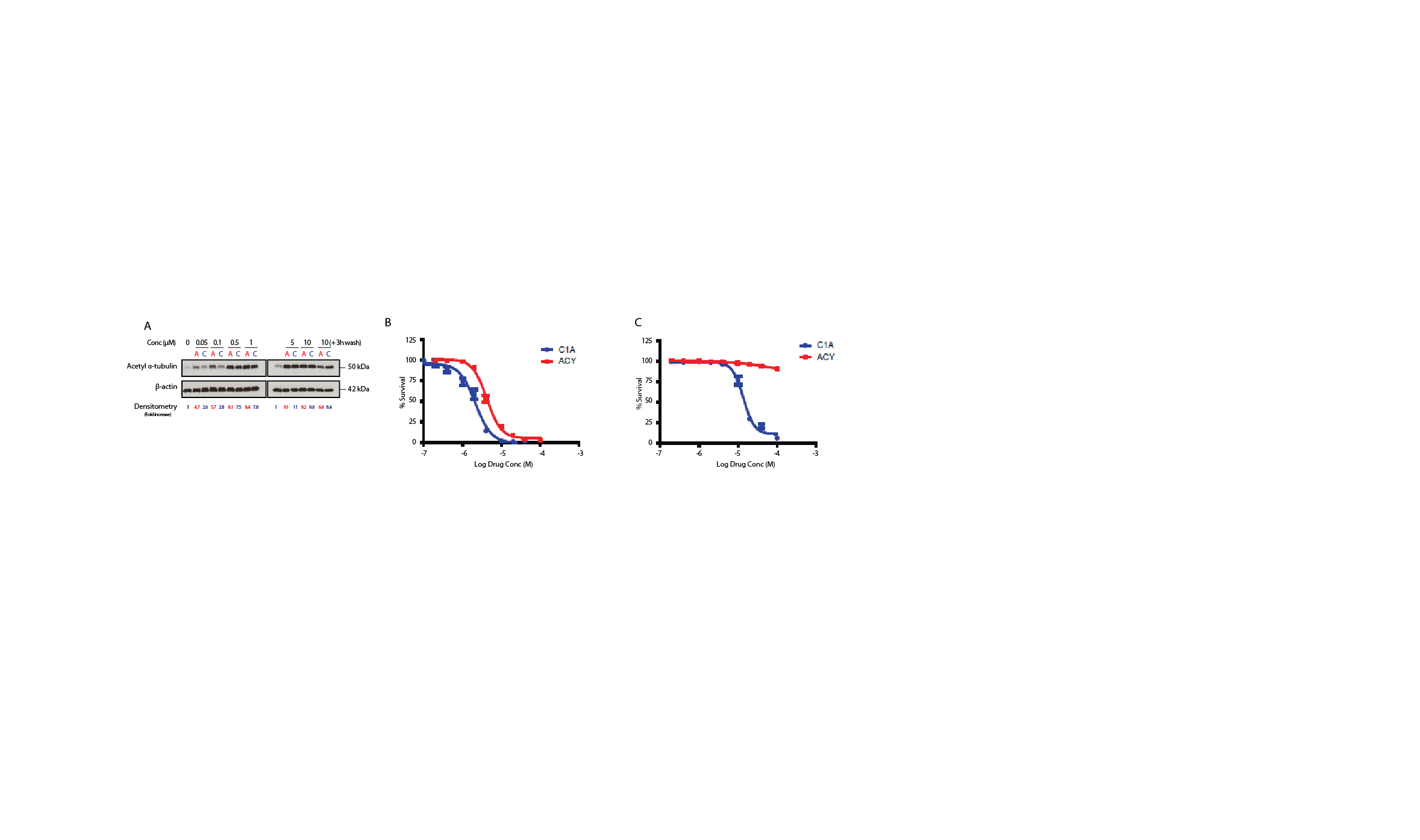
**

**Supplementary Fig 2. C1A is associated with superior activity in HCT116 colon cancer cells following ‘pulsed treatment-drug washout’ when compared to ACY-1215.** **A**. HCT116 cells were treated continuously at the indicated concentration for 1 hour prior to western blotting. Wash out conditions were also tested to compare both compounds; cells were treated for 1 hour at 10 μM, washed with PBS and replenished with full media, allowed additional 3 hours prior to western blotting. Densitometry were normalized between levels of acetyl-tubulin and loading controls (actin) and expressed as fold increase compared to control. (A, ACY-1215; C, C1A) **B**. Growth inhibitory assay of C1A versus ACY-1215 (ACY); HCT-116 cells were continuously treated for 72 hours prior at increasing concentrations. **C**. Growth inhibitory assay of C1A versus ACY-1215 (ACY); HCT116 cells were treated for 6 hours, then washed with PBS and replenished with full media and allowed to grow for further 66 hours. ­


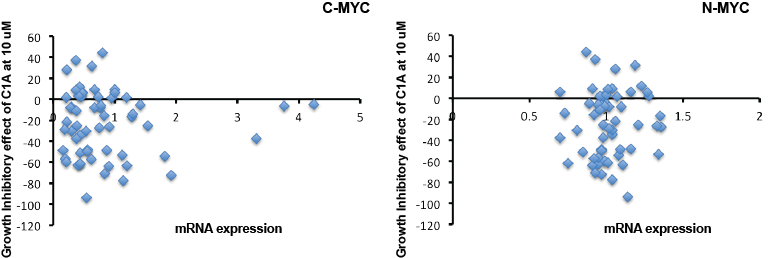


**Supplementary Fig. 3. Relationship between *C-MYC* or *N-MYC* mRNA expression and the total growth inhibition of C1A in the NCI60 panel of cell lines.** The growth inhibitory effect of C1A was tested across the NCI60 panel following 72 h exposure at 10 μM. Data on mRNA levels were from the Developmental Therapeutics Program (NCI/NIH) available online.

**Supplementary Table 1.** Growth inhibitory effect of HDAC6 inhibitors C1A and ACY-1215 and pan-HDAC inhibitor SAHA in panel of Multiple Myeloma and Neuroblastoma cell lines. Cells were treated continuously for 72 h with HDAC inhibitors.

| **Multiple Myeloma** | |  |  |  |  |  |  |  |  |
| --- | --- | --- | --- | --- | --- | --- | --- | --- | --- |
|  |  |  |  |  |  |  |  |  |  |
| **Cell line** |  |  | **C1A** | | |  | **ACY-1215** | | |
|  |  |  |  |  |  |  |  |  |  |
|  |  |  | **GI_50_ (μM)** |  | SD |  | **GI_50_ (μM)** |  | SD |
|  |  |  |  |  |  |  |  |  |  |
| **ARH77** |  |  | 0.30 |  | 0.06 |  | 0.70 |  | 0.49 |
| **JJN3** |  |  | 0.68 |  | 0.05 |  | n.d. |  | n.d. |
| **KMS12** |  |  | 0.35 |  | 0.06 |  | 0.31 |  | 0.10 |
| **U266** |  |  | 0.47 |  | 0.08 |  | 1.33 |  | 0.73 |
| **RPMI-8266** |  |  | 1.18 |  | 0.72 |  | 1.46 |  | 0.06 |
| **KMS11** |  |  | 0.10 |  | 0.03 |  | n.d. |  | n.d. |
| **OPM-2** |  |  | 0.27 |  | 0.15 |  | 1.62 |  | 0.32 |
| **KMS-12BM** |  |  | 0.51 |  | 0.10 |  | 0.81 |  | 0.15 |
|  |  |  |  |  |  |  |  |  |  |
| **Mean** |  |  | 0.48 |  | 0.33 |  | 1.04 |  | 0.51 |
|  |  |  |  |  |  |  |  |  |  |
| **Neuroblastoma** | |  |  |  |  |  |  |  |  |
|  |  |  |  |  |  |  |  |  |  |
| **Cell line** | **Neuroblastic (N)** | | **C1A** | | |  | **SAHA** | | |
|  | **Stromal (S)** |  |  |  |  |  |  |  |  |
|  |  |  | **GI_50_ (μM)** |  | SD |  | **GI_50_ (μM)** |  | SD |
|  |  |  |  |  |  |  |  |  |  |
| **KELLY** | N |  | 0.18 |  | 0.01 |  | 0.40 |  | 0.12 |
| **SH-SY5Y** | N |  | 0.28 |  | 0.00 |  | 0.92 |  | 0.3 |
| **SHEP** | S |  | 16 |  | n.d. |  | 1.4 |  | 0.47 |
| **SKNAS** | S |  | 7.1 |  | 1.5 |  | 0.49 |  | 0.02 |
| **SK-N-BE(2)C** | N |  | 0.66 |  | 0.19 |  | 0.63 |  | 0.08 |
| **IMR32** | N |  | 1.0 |  | 0.21 |  | 0.35 |  | 0.09 |
|  |  |  |  |  |  |  |  |  |  |
| **Mean** |  |  | 4.2 |  | 6.3 |  | 0.69 |  | 0.39 |

­

n.d., not determined.

**References**

Kawaguchi Y, Kovacs JJ, McLaurin A, Vance JM, Ito A, Yao TP (2003) The deacetylase HDAC6 regulates aggresome formation and cell viability in response to misfolded protein stress. *Cell* **115**(6)**:** 727-38

Lee JY, Koga H, Kawaguchi Y, Tang W, Wong E, Gao YS, Pandey UB, Kaushik S, Tresse E, Lu J, Taylor JP, Cuervo AM, Yao TP (2010) HDAC6 controls autophagosome maturation essential for ubiquitin-selective quality-control autophagy. *The EMBO journal* **29**(5)**:** 969-80
